# Preventive Effect of Raw Liubao Tea Polyphenols on Mouse Gastric Injuries Induced by HCl/Ethanol via Anti-Oxidative Stress

**DOI:** 10.3390/molecules23112848

**Published:** 2018-11-01

**Authors:** Yu Qian, Jing Zhang, Xinwei Fu, Ruokun Yi, Peng Sun, Mei Zou, Xingyao Long, Xin Zhao

**Affiliations:** 1 Chongqing Collaborative Innovation Center for Functional Food, Chongqing University of Education, Chongqing 400067, China; qianyubaby@126.com (Y.Q.); yirk@cque.edu.cn (R.Y.); sunpeng@foods.ac.cn (P.S.); zoumei@foods.ac.cn (M.Z.); longyaoyao@foods.ac.cn (X.L.); 2 Chongqing Engineering Research Center of Functional Food, Chongqing University of Education, Chongqing 400067, China; 3 Chongqing Engineering Laboratory for Research and Development of Functional Food, Chongqing University of Education, Chongqing 400067, China; 4 College of Biological and Chemical Engineering, Chongqing University of Education, Chongqing 400067, China; 5 Department of Environmental and Quality Inspection, Chongqing Chemical Industry Vocational College, Chongqing 400067, China; zjinger0810@126.com; 6 College of Life Sciences, Chongqing Normal University, Chongqing 400047, China; fuxinwei@chongq.picc.com.cn; 7 Department of Food Science and Biotechnology, Cha University, Seongnam 13488, Gyeongghi-do, Korea

**Keywords:** Liubao tea, polyphenol, gastric injury, oxidative, protein

## Abstract

Liubao tea is a type of traditional Chinese tea, belonging to the dark teas. This study is a basic research of the contained polyphenols (active substances) and detected preventive effects of polyphenols of raw Liubao tea (PRLT) on mouse gastric injuries induced by HCl/ethanol. High-pressure liquid chromatography was used to analyze the components of PRLT. Furthermore, a mouse gastric injury model was established to observe the preventive effects. PRLT was shown to contain gallic acid, EGC (epigallocatechin), catechin, caffeine, EC (epicatechin), EGCG (epigallocatechin gallate), GCG (gallocatechin gallate), and ECG (epicatechin gallate). The results of the in vivo study indicate that PRLT can inhibit the observed increase of gastric juice volume and decrease of gastric juice pH caused by gastric injury. PRLT can decrease the serum levels of IL-6 (interleukin-6), IL-12 (interleukin-12), TNF-α (tumor necrosis factor-α), and IFN-γ (interferon-γ) in mice with gastric injuries. Moreover, it can also increase the serum levels of SS (somatostatin) and VIP (vasoactive intestinal peptide) and reduce the serum levels of both SP (substance P) and ET-1 (endothelin-1). PRLT was also shown to increase SOD (superoxide dismutase) and GSH (glutathione) levels and decrease MDA (malondialdehyde) level. The detection of mRNA and protein in gastric tissues indicates that PRLT could also up-regulate the expression of Cu/Zn-SOD (copper/zinc superoxide dismutase), Mn-SOD (manganese superoxide dismutase), CAT (catalase), nNOS (neuronal nitric oxide synthase), and eNOS (endothelial nitric oxide synthase) and down-regulate the expression of both iNOS (inducible nitric oxide synthase) and COX-2 (cyclooxygenase-2). Thus, PRLT possess a good preventive effect on gastric injury, which is directly related to the contained active substance. PRLT show good anti-oxidative and preventive effect in gastric injury and offer promising application value.

## 1. Introduction

Liubao tea is a famous tea with a long history in China; its name is derived from the place of origin: Liubao town of Guangxi Province [1]. The appearance of Liubao tea is black brown with a flaky shape. The picked tea is cooked into green and placed into bamboo baskets without rolling or compression, thus leaving the tea in the most natural status, as an unfermented tea. Fermented Liubao tea has a rope strip shape [2]. Some is further processed into tea cakes and tea bricks via compacting, and some is rolled and compressed into large and small baskets, allowing slow natural fermentation. It has been reported that the tea polyphenols, flavonoids, caffeine, free amino acids, and soluble saccharides in Liubao tea possess many positive effects, such as lipid reduction, regulation of the glucolipid metabolism, antioxidation, regulation of immune function, and regulation of the intestinal flora [3,4,5,6].

About 10% of all people suffer from peptic ulcer worldwide. With the increase in the incidence and morbidity, peptic ulcer has gradually developed to one of the most severe gastrointestinal tract diseases worldwide. Under normal status, an important equilibrium exists between secreted gastric acid and the gastroduodenal defense. The equilibrium may be broken by specific factors, including anti-inflammatory drugs, *Helicobacter pylori*, bile salt, acid, alcohol, and pepsin. All these factors cause gastric mucosa injuries, or gastric ulcers when severe. Patients with peptic ulcer often suffer from gastroduodenal bleeding, perforation, and obstruction, or even die when the case is severe. The ethanol entering into the body will disorder the electron transport system in mitochondria, increase reactive oxygen content, and lead to cellular injury. The reactive oxygen can promote the myeloperoxidase activity in gastric tissues and damage gastric mucosa. All these reactive oxygen species (ROS) can also cause the release of other inflammatory mediators in large quantities. Furthermore, the increase in neutrophils could aggravate the inflammatory response and gastric ulcers [7].

Tea polyphenols are very important component in tea and exert a significant effect on eliminating ROS. During the peroxidation process, the tea polyphenols will occupy the generated lipid peroxide free radicals, generate polyphenol free radicals with low activity, and terminate the free radical oxidation chain reaction. Moreover, the activities of multiple antioxidant enzymes (such as SOD, GSH-Px, and CAT) can be increased by tea polyphenols, which can highly efficiently eliminate free radicals [8]. It has been reported that tea polyphenols exert protective effects on alcoholic gastric injury, chemical gastric injury, and acute and chronic gastritis [9,10,11]. They can also inhibit precancerous lesions of gastric cancer and the growth of cancer cells [12]. However, although few tea polyphenol monomers have been shown to exert a protective effect on gastric injury, reports on the gastric protection and component analysis of tea polyphenols are still limited. Especially, a Liubao tea-related investigation has not been conducted.

The health of the gastrointestinal tract is very important for the maintenance of a high quality of life. Digestive absorption, metabolism, and mucosal immunity of the gastrointestinal tract decline with age (as well as gastric mucosa) [13]. While gastritis, among the chronic gastric diseases, may not turn into gastric cancer, the possibility exists. Repeated gastritis caused by gastric injury, repair, and re-repair of gastric mucosa will lead to intestinal metaplasia in the stomach. Intestinal metaplasia is often considered as a precancerous lesion and will turn into early gastric cancer without further intervention. The protection by food, especially functional food with active ingredients has become a research hotspot. Tea polyphenols have been proved to exert inhibitory effects on a variety of inflammation-related diseases [14,15]. Investigations involving interleukin on the inflammatory inhibition are increasingly conducted.

Liubao tea is a traditional dark tea. However, due to limited research and development, its value has not been fully accessed. Other renowned teas have been fully investigated, including the extraction of functional components and product development. In this study, the preventive effects of PRLT on gastric injury were investigated. Moreover, a biological molecular detection method was used to explore the mechanism, providing a theoretical basis for the development of a high-class Liubao tea resource.

## 2. Results

### 2.1. Analysis of PRLT Component

**Figure 1:**
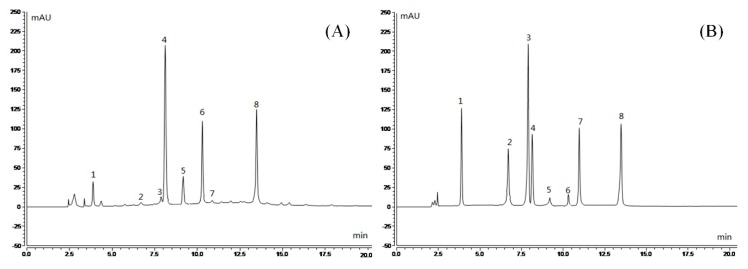
Polyphenol constituents of polyphenols of raw Liubao tea (PRLT). (**A**) Standard chromatograms; (**B**) PRLT chromatograms. 1: gallic acid; 2: EGC (epigallocatechin); 3: catechin; 4: caffeine; 5: EC (epicatechin); 6: EGCG (epigallocatechin gallate); 7: GCG (gallocatechin gallate); and 8: ECG (epicatechin gallate).

40 μL of each of 21 stock solutions were mixed to prepare standard mixed solution. Under the above chromatographic condition, when the equipment was stable, the mixed solution was detected. Twenty-one single standard solutions were prepared by diluting standard stock solutions (20 μL) 20 times and detected using the same chromatographic condition. Seven polyphenols (gallic acid, EGC, catechinic acid, EC, EGCG, GCG, and ECG) and one alkaloid (caffeine) were confirmed after comparison with the chromatograms of test samples (Figure 1). The contents of each component in PRLT were calculated using an external standard method, as shown in Table 1.

### 2.2. Influence of PRLT on Volume and pH of Gastric Juice

As shown in Table 2, compared to the normal group, the gastric juice secretion volume in the control group after gastric injury increased significantly, and the secretion volumes in the PRLT-L group, PRLT-H group, and ranitidine group were lower than in the control group. The effect in the PRLT-H group was close to that of the ranitidine group. Furthermore, compared to the normal group, the pH of the gastric juice in the control group decreased significantly. Except for the normal group, pH in the other three groups were increased compared to the model group. The increase in pH in the PRLT-H group was close to that of the ranitidine group.

### 2.3. PRLT after Gastric Mucosa Injury

**Figure 2:**
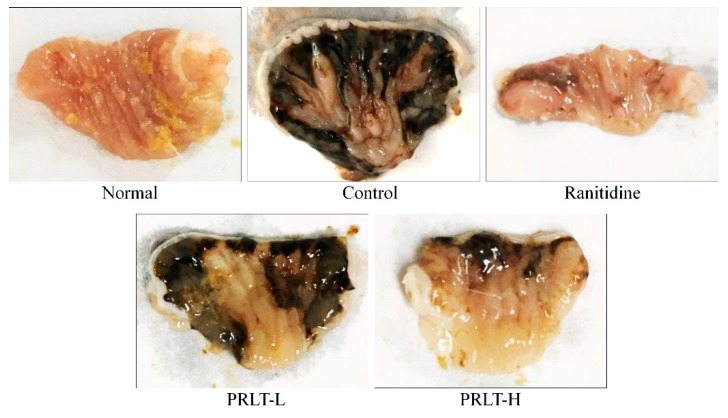
Morphological observation of gastric injury in experimental mice. PRLT-L: 100 mg/kg polyphenols of raw Liubao tea treatment; PRLT-H: 200 mg/kg polyphenols of raw Liubao tea treatment; and ranitidine: 50 mg/kg ranitidine treatment.

As shown in Figure 2 and Table 3, the area of gastric mucosa injury in the control group was the largest. The treatment in the PRLT-L and PRLT-H group significantly (*p* < 0.05) decreased the area, and ranitidine had the highest inhibition rate on the injury area. The effect of PRLT-H was close to that of ranitidine, which was significantly higher than that of PRLT-L (*p* < 0.05). Thus, PRLT could effectively reduce the influence of gastric injury on gastric mucosa.

### 2.4. Gastric Histopathology Observation

**Figure 3:**
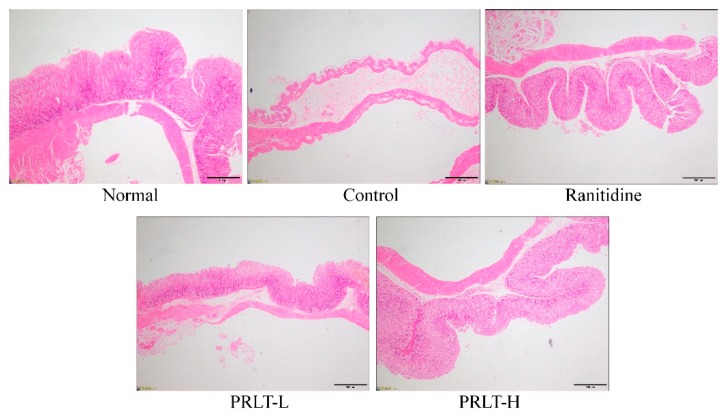
Histopathological observation of gastric tissue in experimental mice (40×). PRLT-L: 100 mg/kg polyphenols of raw Liubao tea treatment; PRLT-H: 200 mg/kg polyphenols of raw Liubao tea treatment; and ranitidine: 50 mg/kg ranitidine treatment.

As illustrated in Figure 3, compared to the normal group, the gastric mucosa in the control group showed significant gastric injury. The intercellular space was significantly increased, and the gastric injury was the most severe. After PRLT treatment, gastric mucosa injury conditions in the normal group and ranitidine group were not severer than in the model groups. The injury condition in the PRLT-H group was the smallest except for the normal group and the effect was superior to that of the PRLT-L group. Thus, raw tea polyphenols in Liubao tea exert a protective effect on the gastric injury to a certain degree, and the effect is better at high concentrations.

### 2.5. Serum SS, SP, VIP, and ET-1 Levels

As shown in Table 4, serum SS and VIP levels in the normal group were highest, while both SP and ET-1 levels were the lowest, showing opposite tendency to the control group. After PRLT treatment, the serum SS and VIP levels in mice with gastric injury were significantly increased (*p* < 0.05), and the SP and ET-1 levels were significantly decreased (*p* < 0.05). The capability of PRLT for regulating serum SS, SP, VIP, and ET-1 to the normal levels was slightly lower than that of ranitidine; however, the effect of PRLT at high concentration was stronger than that at low concentration.

### 2.6. Serum IL-6, IL-12, TNF-α, and IFN-γ Levels

As shown in Table 5, serum IL-6, IL-12, TNF-α, and IFN-γ levels in the control group were highest; however, those in the control group were lowest. After treatment with raw tea polyphenols and ranitidine, serum IL-6, IL-12, TNF-α, and IFN-γ levels in mice with gastric injury decreased compared to the control group. The decreasing amplitude in the ranitidine group was highest, and the decreasing amplitude in the PRLT-H group was also higher than in the PRLT-L group.

### 2.7. SOD, GSH, and MDA Levels in Serum and Gastric Tissues

As shown in Table 6, SOD and GSH levels in mouse serum and gastric tissues were highest in the normal group, while the MDA level is the lowest. The SOD and GSH levels after gastric injury induced by HCl/ethanol decreased, while MDA level increased. Ranitidine, PRLT-H, and PRLT-L could significantly (*p* < 0.05) inhibit the decrease in GSH and SOD levels and increase in MDA level in gastric tissues and serum in the gastric injury mice induced by HCl/ethanol. The effect of ranitidine was strongest and the effect of PRLT-H was also stronger than that of PRLT-L.

### 2.8. mRNA and Protein Expression of Cu/Zn-SOD, Mn-SOD, and CAT mRNA in Gastric Tissues

**Figure 4:**
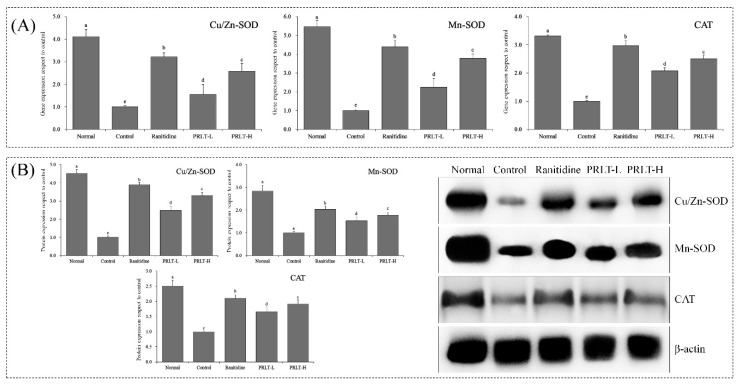
Cu/Zn-SOD, Mn-SOD and CAT mRNA (**A**) and protein (**B**) expression in gastric tissues of mice. Values presented are the mean ± standard deviation (N = 3/group). ^a–^^e^ Mean values with different letters in the same bars are significantly different (*p* < 0.05) according to Duncan’s multiple-range test. PRLT-L: 100 mg/kg polyphenols of raw Liubao tea treatment; PRLT-H: 200 mg/kg polyphenols of raw Liubao tea treatment; Ranitidine: 50 mg/kg ranitidine treatment. Cu/Zn-SOD: copper/zinc superoxide dismutase; Mn-SOD: manganese superoxide dismutase; and CAT: catalase.

As illustrated in Figure 4, the expression of mRNA and protein of Cu/Zn-SOD in the gastric tissues in the normal group was the highest, while SOD1 expression in the control group was the lowest. PRLT-H and PRLT-L could both up-regulate Cu/Zn-SOD expression, and the up-regulation capability was lower than that of ranitidine. Furthermore, the expressions of mRNA and protein of Mn-SOD were 5.46 and 3.12-fold that of the corresponding expression in the control group. The expression levels of mRNA and protein in the PRLT-H group were 3.78 and 3.02-fold that of those in the control group, which is slightly lower than the ranitidine group but higher than the PRLT-L group. The detection of CAT mRNA and protein also indicated that the CAT expression in the gastric tissues in the normal group was significantly stronger (*p* < 0.05) than those in the ranitidine group, PRLT-H group and PRLT-L group. PRLT showed the ability of down-regulating CAT expression.

### 2.9. mRNA and Protein Expression of nNOS, eNOS, and iNOS in Gastric Tissues

**Figure 5:**
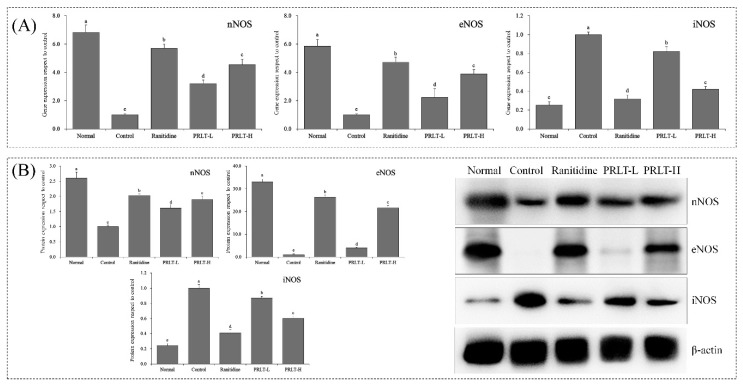
nNOS, eNOS and iNOS mRNA (**A**) and protein (**B**) expression in gastric tissues of mice. Values presented are the mean ± standard deviation (N = 3/group). ^a–^^e^ Mean values with different letters in the same bars are significantly different (*p* < 0.05) according to Duncan’s multiple-range test. PRLT-L: 100 mg/kg polyphenols of raw Liubao tea treatment; PRLT-H: 200 mg/kg polyphenols of raw Liubao tea treatment; Ranitidine: 50 mg/kg ranitidine treatment. nNOS: neuronal nitric oxide synthase; eNOS: endothelial nitric oxide synthase; and iNOS: inducible nitric oxide synthase.

As shown in Figure 5, mRNA and protein expression of nNOS in the gastric tissues in the control group were the lowest. The mice in the normal group showed the highest nNOS expressions of 6.82 and 3.89-fold of that of the control group. Both the mRNA and protein expression levels of nNOS in the gastric tissues of PRLT-L group achieved 4.55 and 3.77-fold of that of the control group, which was significantly higher than the PRLT-L group (*p* < 0.05), but lower than the ranitidine group. The results showed that the mRNA and protein expression of eNOS in the gastric tissues in the normal group, PRLT-L group, PRLT-H group, and ranitidine group were 5.85, 2.23, 3.89, and 4.72-fold, and 5.12, 2.45, 3.12, and 4.33-fold of that of the control group, respectively. In contrast, iNOS expression in the gastric tissues of the control group was the highest. After PRLT and ranitidine treatment, PRLT-L, PRLT-H, and ranitidine could down-regulate the iNOS expression level 0.82, 0.42, and 0.32-fold, and 0.75, 0.25, 0.12-fold in the control group, respectively, which was significantly higher than in the normal group.

### 2.10. mRNA and Protein Expression of COX-2 in Gastric Tissues

**Figure 6:**
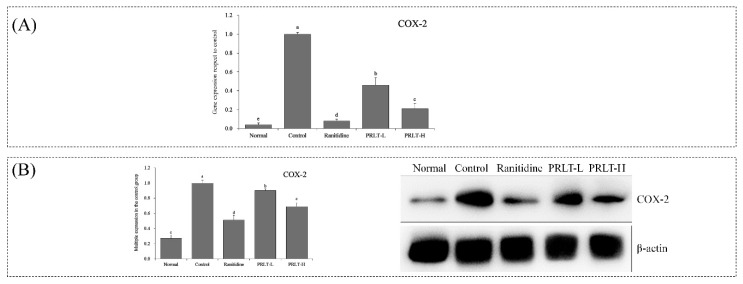
COX-2 mRNA (**A**) and protein (**B**) expression in gastric tissues of mice. Values presented are the mean ± standard deviation (N = 3/group). ^a–^^e^ Mean values with different letters in the same bars are significantly different (*p* < 0.05) according to Duncan’s multiple-range test. PRLT-L: 100 mg/kg polyphenols of raw Liubao tea treatment; PRLT-H: 200 mg/kg polyphenols of raw Liubao tea treatment; and ranitidine: 50 mg/kg ranitidine treatment. COX-2: cyclooxygenase-2.

As shown in Figure 6, mRNA and protein expression of COX-2 in the gastric tissues are significantly lower than other groups (*p* < 0.05), which is 0.04-fold and 0.08-fold that of the control group. The mRNA and protein expression of COX-2 in the gastric tissues of PRLT-L, PRLT-H, and ranitidine groups was also significantly lower than that of the control group (*p* < 0.05).

## 3. Discussion

Gastric injury not only severely damages the stomach, but also injuries the blood, digestive system, and nervous system at different degrees. PRLT were selected in this study, and the protective effect and mechanism of PRLT on the gastric injury was explored via animal experiment. When the gastric mucosa is injured, the gastric juice volume increases correspondingly, while the pH in the gastric juice will decrease simultaneously [16]. The results indicate that PRLT could alleviate the influence of gastric injury on gastric juice, and PRLT at high concentration achieved better efficacy. Photographs of gastric mucosa and H&E sections under microscope were the methods used to observe the protective effect of the active substance on gastric injury [17], by which PRLT has been shown to alleviate the injury of gastric tissues.

After inflammation, proinflammatory cytokines increase, which are closely related with development of acute gastric ulcers. Injuries of cells and organs will be aggravated with increasing proinflammatory cytokines. IL-6 can promote proliferation and differentiation and is closed related to various forms of inflammation. IL-6 is a chemokine for monocyte and inflammatory cells, leading to abundant generation and release of inflammatory mediators, damage of the gastric mucosa barrier, gastric mucosa injury, and aggravation of inflammation [18]. IL-12 is a type of factor that is closely related to inflammation, and plays a very important role in immunoregulation. Increase in IL-12 expression is also a manifestation of inflammation aggravation [19]. IFN-γ is a cytokine that is generated by activated T cells and NK cells, and possesses multiple biological effects. It has significant effects in ulcer tissues, and has a positive correlation with the injury degree of gastric mucosa cells [20]. Similar to IFN-γ, TNF-α exerts positive effects on gastric mucosa, including causing neutrephil infiltration and the collapse of cytoskeleton. It can also lead to a release of many free radicals and proinflammatory cytokines, thus greatly aggravating organ injury and damaging cell structure; therefore, the gastric tissues are severely injured [21]. The serum IL-6, IL-12, TNF-α, and IFN-γ levels after gastric injury greatly increased. After PRLT action, the proinflammatory cytokine levels decreased. The effect is close to that of ranitidine. Thus, PRLT are active substances with inflammatory inhibitory effect.

SS widely exists in body, and also in the intestinal plexus and stomach, while being less prevalent in both gastric juice and intestinal juice. SS possesses an inhibition effect on peristole and decreases the blood flow volume in the gastrointestinal tract wall. When the stomach is in discomfort, it inhibits the secretion of gastric acid [22]. SP can promote the secretion of gastric acid and pepsin, lead to gastrointestinal contraction, increase the blood flow volume in the stomach, and aggravate gastric injury [23]. VIP can promote SS release from D cells, can further inhibit the secretion of gastric acid, and plays a positive role in the gastric injury [24]. When gastric injury happens, VIP level and SS secretion decline, leading to accelerated secretion of gastric acid and high gastric mucosa injury degree [25]. ET-1 is a vasoconstrictive active substance, and can cause anoxia and acidosis in the stomach under stress. Under such a situation, the generation of large amounts of free radicals will promote the release of abundant ET-1. Such unfavorable circulation aggravates the injury of gastric mucosa and leads to gastric function disorder [26]. PRLT inhibit the secretion of gastric acid, reduce pH in gastric juice, and protect gastric mucosa by increasing serum SS and VIP levels, while decreasing SP and ET-1 levels.

After inflammation, free radicals (ROS and RNS) rapidly aggregate, which will further aggravate the injury and toxic reaction in gastric tissue, and the gastric injury degree will greatly increase [27]. It has been reported that after gastric injury, the levels of GSH and SOD will decrease, while the MDA level will significantly increase [28]. In this study, PRLT significantly influenced SOD, GSH, and MDA levels in mice with gastric injury, while regulation of their levels could protect the injury.

SOD is very important to balance antioxidant and oxidant systems, which can effectively eliminate oxygen radicals, and protect cells from oxidative injury [29]. SOD1 (Cu/Zn-SOD), an isomer of SOD, exists in the cell plasma. It is a free-radical scavenger of SOD that uses Cu^2+^ and Zn^2+^ as activity centers. SOD1 can eliminate the toxic effect caused by O^2−^, and thus protect gastric tissues [30]. SOD2 (Mn-SOD), which is also an isomer of SOD and a SOD free-radical scavenger, exists in mitochondria and uses Mn^4+^ as an activity center. Gastric tissues contain many mitochondria, in which the activity of SOD2 is significantly decreased after HCl/ethanol induced liver injury. The results are consistent with those found in the presented study [31]. As an important antioxidant enzyme, CAT can eliminate hydrogen peroxide in the body, inhibit oxidative stress, alleviate oxidation caused by HCl/ethanol, and inhibit gastric injury [32]. Furthermore, the synergistic effect between CAT and SOD can accelerate the elimination of free radicals [6]. Thus, PRLT exert a good antioxidant effect, inhibit gastric injury by regulating SOD1, SOD2, and CAT expression in gastric tissues, and protect gastric mucosa.

nNOS belongs to the nervous type NOS and exerts a protective effect on nerve cells. The distribution in gastric mucosa is wider than in both eNOS and iNOS. nNOS widely exists in endocrine cells, and coordinates with gastrointestinal hormones, thus regulating gastrointestinal function. It can effectively protect gastric mucosa and positively affect the repair of injured gastric mucosa [33]. eNOS is an endothelial type NOS that is stably expressed in gastric mucosa. NO generated by eNOS can promote the repair of gastric mucosa and the regulation of the blood flow volume in gastric mucosa. Furthermore, eNOS can also inhibit the secretion of gastric acid, improve the barrier function of mucus, and promote the regeneration of vessels, playing a role in repairing gastric mucosa injury [34]. iNOS is an inducible type of NOS. As a rate-limiting enzyme during NO synthesis, NOS is abundant in normal tissues. After iNOS activation, the enzyme activity can sustain for an extended period of time, and large amounts of NO will be released. NO with low concentration exerts an inhibitory effect on gene mutation to a certain degree. Moreover, it can improve the defense capability. However, excessive NO will lead to control disorders of gene mutation, activation of gene mutation, and lesion in tissues [35]. Increased iNOS expression can be found at inflammatory sites, activating the generation of an abundance of inflammatory mediators by iNOS and leading to an aggravation of gastric injury [36]. PRLT can up-regulate nNOS and eNOS expression, down-regulate iNOS expression, control inflammation development, and repair gastric mucosa injury.

COX-2 is an isoenzyme of COX that rapidly stimulates induced reactions with a series of injury chemical factors, participates in the inflammatory response, and promotes inflammation development. Thus, when the cells are stimulated by inflammation, COX-2 will be expressed abundantly [37]. Furthermore, iNOS has a synergistic effect with COX-2, and can further improve inflammatory response [38]. The PRLT can down-regulate COX-2 expression, exerting an anti-inflammatory effect and reducing gastric injury.

Gallic acid (GA) has been reported to have many biological activities, such as anti-inflammation, antimutation, and antioxidation [39]. As a polyphenol, GA has strong antioxidant and anti free radical effects. GA has also been reported to exert a scavenging effect on superoxide anion free radicals, reduce the accumulation of ROS in histocytes, and inhibit inflammation via antioxidant effects [40]. The results obtained in this study indicate that catechinic acid, EGC, EC, EGCG, GCG, and ECG all exist in PRLT as catechins with strong antioxidant effects. The reducibility of EGCG is 100-fold that of L-erythorbic acid [41]. It has been reported that the resistance to oxidation of catechins is higher than vitamin E and has synergistic effects with both vitamin C and E [42]. Catechinic acid can improve immunity via antioxidant effects [43] and has been reported to adhere on the gastric wound, thus protecting the ulcer wound surface [44]. Catechinic acid exerts has various effects, such as reducing the permeability of blood capillary, hemostasis, killing fungus, and prevention of gastric ulcers [45]. In this study, PRLT also showed antioxidant effects and inhibition effects on gastric injury in vivo. Caffeine is often detected during the extraction of tea polyphenols. As an alkaloid, caffeine can decrease oxidative stress [46]. Clinical research also indicates that caffeine can control the quantity of some inflammatory molecules and inhibit inflammation [47]. PRLT play an antioxidant role in mice with gastric injury through these active substances or synergistic effects, further forming a protective system of gastric tissue, thus protecting from gastric injury.

## 4. Materials and Methods

### 4.1. PRLT Extraction

Metal ion precipitation was used to extract polyphenols of raw Liubao tea (PRLT). Plant polyphenols can form crystalline precipitation with metal ions that can be extracted from extraction liquid to realize preliminary and fast separation. Then, a pure polyphenol extract solution can be obtained via dissolution with acid and extraction with ethyl acetate. The tea polyphenol extractive is finally collected via vacuum drying [48]. The procedures were as follows: 100 g raw Liubao tea were weighted and ground, 150 mL 45% ethanol solution was added to the powder, and the mixture was immersed for 30 min at 90 °C. The above procedure was repeated once, and both solution were combined after immersion, and the pH was adjusted to 6.0. Then, 160 mL AlCl_3_ (6 g) and ZnCl_2_ (12 g) were added to precipitate PRLT. The mixture was centrifuged at 3000 rpm/min for 10 min and 200 mL HCl (12%) was added to dissolve the collected precipitate. The supernatant was separated and extracted twice with 200 mL of ethyl acetate. Finally, the polyphenol extract was obtained via rotary evaporation [49].

### 4.2. Analysis of the PRLT Component

The standard substance was weighted (10 mg, dried under reduced pressure at 25 °C for 24 h) to an accuracy of 0.01 mg. The standard substance was dissolved in methanol in a 10 mL volumetric flask to a volume of 10 mL. Furthermore, 12.5 mg polyphenol extractive was weighted and placed into a 25 mL volumetric flask. The extractive was dissolved in methanol, and well shaken to prepare the test solution (0.5 mg/mL). The chromatographic conditions were as follows: DAD detector; pentafluobenyl column (150 mm × 2.1 mm, 2.6 μm); mobile phase A: 0.1% formic acid, mobile phase B: acetonitrile; flow rate: 0.6 mL/min; temperature: 30 °C; injection volume: 10 μL; wave length: 280 nm; the gradient elution condition is shown in Table 7.

### 4.3. Animal Experiment

Kunming (KM) mice are outbreeding mice, they came from swiss mice. SPF (specific pathogen free) KM mice (six weeks old, male) were obtained from the Laboratory Animal Center, Chongqing Medical University, and raised at a temperature of 22 ± 4 °C and a humidity of 50 ± 20%. The mice were randomly divided into five groups (n = 10): normal group, control group, low-dose PRLT administrated by gavage group (PRLT-L group, 100 mg/kg), high-dose PRLT administrated by gavage group (PRLT-H group, 200 mg/kg), and ranitidine administrated by gavage group (50 mg/kg). After a one-week acclimation, normal food and drink were allowed for the mice in the normal group and control group. PRLT solutions at 100 mg/kg (0.2 mL) and 200 mg/kg (0.2 mL) were administrated by gavage in the PRLT-L group and PRLT-H group, respectively. Ranitidine solution at 50 mg/kg (0.2 mL) was administrated by gavage in the ranitidine group. The doses were given for two weeks. At Day 14, the mice were not allowed to eat but had free access to water. After fasting for 24 h, except for the mice in the normal group, other mice were administrated with inducer (60% ethanol and 40% 150 mmol/HCl) by gavage. After 30 min, all mice were dissected to collect gastric juices and gastric tissues. The eyeballs were extracted using a tweezer [22]. The gastric area was photographed, and the injury area was measured via ImageJ, based on which the inhibition rate of gastric injury by PRLT was calculated. This study was approved by the Animal Ethics Committee of Chongqing University of Education (Chongqing, China), Laboratory animal using license No. SYXK (Yu) 2018-0003.

### 4.4. Detection for Serum ET-1, SS, SP, and VIP Levels

The blood was separated to obtain serum (4500 rpm/m, 15 min), in which ET-1, SS, SP, and VIP levels were detected in kits (Abcam, Cambridge, MA, USA) [50].

### 4.5. Detection for Serum IL-6, IL-12, TNF-α, and IFN-γ Levels

The serum was obtained as above. Serum levels of IL-6, IL-12, TNF-α, and IFN-γ were detected using kits (Abcam, Cambridge, MA, USA).

### 4.6. Detection for MDA Level in SOD and GSH from Serum and Gastric Tissue

The serum was obtained as above. Colon tissues and saline were mixed at a ratio of 1:9, and ultrasonicated into tissue homogenate. SOD, GSH, and MDA levels in serum and gastric tissues were detected using kits (Nanjing Jiancheng Bioengineering Institute, Nanjing City, China) [50].

### 4.7. qPCR Assay

Gastric tissues were ground. Total RNA was extracted using RNAzol, and diluted into one μg/μL. 5 μL of the diluted total RNA solution was reverse transcribed to obtain cDNA template. two μL cDNA template was mixed with 10 μL SYBR Green PCR Master Mix and 1 μL up-stream and down-stream primers (Table 8), at 95 °C for 60 s and 40 cycles: 95 °C, 15 s; 55 °C, 30 s; 72 °C, 35 s; 95 °C, 30 s; and 55 °C, 35 s for detection using GAPDH as an internal reference. The relative expression quantity was calculated using the 2^−ΔΔCt^ method [50].

### 4.8. Western Blot Analysis

100 mg of gastric tissue was homogenized with one mL RIPA, 10 µL PMSF, and centrifuged at 12,000 rpm/min under 4 °C for five min. The middle protein layer was used to measure the protein concentration via BCA. The samples were diluted into 50 µg/mL, mixed with Sample Buffer at a ratio of 4:1, heated at 100 °C for five min, prepared into separation gel and stacking gel mixed with mixing acrylamide, resolving buffer, stacking buffer, distilled Water, 10% APS, and TEMED at a specific ratio. A pre-stained protein ladder and the samples were injected into sample wells. Vertical gel electrophoresis was performed in SDS-PAGE loaded with proteins for 50 min. After activation by methanol for one min, the PVFG membrane was transferred, and sealed with TBST containing 5% skim milk for one h. After sealing, the PVDF membrane was washed with TBST, incubated at 25 °C for two h with primary antibody. Then, the membrane was washed with TBST, incubated at 25 °C for one h with secondary antibody. Supersignal West Pico PLUS was spread on the PVDF membrane for observation via iBright FL1000 [51].

### 4.9. Statistical Analysis

All the experiment data showed variance equality and normal distribution. Additionally, the results of triplicates were averaged and analyzed with SAS9.1 using one-way ANOVA. *p* < 0.05 was assumed to indicate a significant difference.

## 5. Conclusions

PRLT have been shown to contain gallic acid, EGC, catechinic acid, caffeine, EC, EGCG, GCG, and ECG via analysis of raw tea polyphenol components. The results of an in vivo experiment indicate that PRLT alleviated the influence of HCl/ethanol on gastric tissues. The results of molecular biological detection demonstrated that PRLT exerted a good antioxidant effect in vivo. The effect can regulate the inflammation-associated cytokines in vivo, regulate body balance, and alleviate gastric injury. Thus, PRLT possess a good protective effect on gastric injury, which is positively correlated to concentration. The effect directly correlates with the active components. PRLT is a type of bioactive substance with development value, which requires deep exploration and development.

## Figures and Tables

**Table 1:** Component content of polyphenols of raw Liubao tea (PRLT).

	C_R_ (mg/L)	A_R_ (mAU*min)	A_X_ (mAU*min)	C_X_ (mg/L)
Gallic acid	24.00	6.7469	2.4674	9.00
EGC	52.00	0.4357	0.3322	40.00
Catechin	21.00	1.6564	0.5913	7.00
EC	50.00	77.5151	3.0796	2.00
EGCG	50.00	35.1070	8.2827	12.00
GCG	45.00	3.1497	0.2794	4.00
ECG	62.00	13.5090	9.6461	44.00
Caffeine	90.00	67.7504	20.5444	27.00

EGC, epigallocatechin; EC, epicatechin; 6: EGCG, epigallocatechin gallate; GCG, gallocatechin gallate; and ECG, epicatechin gallate.

**Table 2:** Gastric juice volume and pH value of gastric acid in experimental mice.

Group	Gastric Juice Volume (mL)	pH Value of Gastric Acid
Normal	0.034 ± 0.008 ^e^	4.700 ± 0.024 ^a^
Control	0.249 ± 0.017 ^a^	1.602 ± 0.043 ^e^
Ranitidine	0.089 ± 0.009 ^d^	4.012 ± 0.020 ^b^
PRLT-L	0.183 ± 0.019 ^b^	2.333 ± 0.023 ^d^
PRLT-H	0.121 ± 0.020 ^c^	3.429 ± 0.026 ^c^

Values presented are the mean ± standard deviation (N = 10/group). ^a–^^e^ Mean values with different letters in the same row are significantly different (*p *< 0.05) according to Duncan’s multiple-range test. PRLT-L: 100 mg/kg polyphenols of raw Liubao tea treatment; PRLT-H: 200 mg/kg polyphenols of raw Liubao tea treatment; and ranitidine: 50 mg/kg ranitidine treatment.

**Table 3:** Gastric injury area and inhibition rate of gastric injury in experimental mice.

Group	Gastric Injury Area (cm^2^)	pH Value of Gastric Acid
Normal	0.00 ± 0.00 ^e^	4.700 ± 0.024 ^a^
Control	1.25 ± 0.31 ^a^	1.602 ± 0.043 ^e^
Ranitidine	0.18 ± 0.05 ^d^	4.012 ± 0.020 ^b^
PRLT-L	0.88 ± 0.26 ^b^	2.333 ± 0.023 ^d^
PRLT-H	0.33 ± 0.09 ^c^	3.429 ± 0.026 ^c^

Values presented are the mean ± standard deviation (N = 10/group). ^a–^^e^ Mean values with different letters in the same row are significantly different (*p* < 0.05) according to Duncan’s multiple-range test. PRLT-L: 100 mg/kg polyphenols of raw Liubao tea treatment; PRLT-H: 200 mg/kg polyphenols of raw Liubao tea treatment; Ranitidine: 50 mg/kg ranitidine treatment.

**Table 4:** Serum levels of SS, SP, VIP and ET-1 in mice.

Group	SS (μg/L)	SP (μg/L)	VIP (μg/L)	ET-1 (μg/mL)
Normal	144.60 ± 5.21 ^a^	69.66 ± 4.02 ^e^	112.65 ± 5.16 ^a^	56.87 ± 3.15 ^e^
Control	42.05 ± 1.89 ^e^	165.78 ± 5.12 ^a^	34.01 ± 2.77 ^e^	125.30 ± 5.56 ^a^
Ranitidine	121.56 ± 3.69 ^b^	106.19 ± 2.91 ^d^	101.29 ± 2.99 ^b^	66.36 ± 2.71 ^d^
PRLT-L	75.20 ± 3.17 ^d^	132.05 ± 4.39 ^b^	74.36 ± 3.13 ^d^	103.64 ± 4.03 ^b^
PRLT-H	105.82 ± 4.36 ^c^	116.18 ± 3.49 ^c^	91.71 ± 3.92 ^c^	75.29 ± 3.37 ^c^

Values presented are the mean ± standard deviation (N = 10/group). ^a–^^e^ Mean values with different letters in the same row are significantly different (*p* < 0.05) according to Duncan’s multiple-range test. PRLT-L: 100 mg/kg polyphenols of raw Liubao tea treatment; PRLT-H: 200 mg/kg polyphenols of raw Liubao tea treatment; Ranitidine: 50 mg/kg ranitidine treatment. SS: somatostatin; SP: substance P; VIP: vasoactive intestinal peptide; and ET-1: endothelin-1.

**Table 5:** Serum cytokines levels of IL-6, IL-12, TNF-α and IFN-γ in mice.

Group	IL-6 (pg/mL)	IL-12 (pg/mL)	TNF-α (pg/mL)	IFN-γ (pg/mL)
Normal	41.08 ± 3.31 ^e^	189.75 ± 15.25 ^e^	35.21 ± 2.62 ^e^	24.65 ± 2.39 ^e^
Control	187.20 ± 6.33 ^a^	765.33 ± 31.08 ^a^	152.71 ± 8.32 ^a^	138.10 ± 6.82 ^a^
Ranitidine	78.26 ± 3.06 ^d^	267.12 ± 20.86 ^d^	51.87 ± 3.98 ^d^	39.71 ± 2.88 ^d^
PRLT-L	135.74 ± 5.21 ^b^	535.69 ± 21.88 ^b^	122.87 ± 6.91 ^b^	101.18 ± 5.31 ^b^
PRLT-H	84.65 ± 4.11 ^c^	318.78 ± 22.53^c^	63.24 ± 4.87 ^c^	52.83 ± 4.37 ^c^

Values presented are the mean ± standard deviation (N = 10/group). ^a–^^e^ Mean values with different letters in the same row are significantly different (*p* < 0.05) according to Duncan’s multiple-range test. PRLT-L: 100 mg/kg polyphenols of raw Liubao tea treatment; PRLT-H: 200 mg/kg polyphenols of raw Liubao tea treatment; Ranitidine: 50 mg/kg ranitidine treatment. IL-6: interleukin-6; IL-12: interleukin-12; TNF-α: tumor necrosis factor-α; and IFN-γ: interferon-γ.

**Table 6:** Levels of SOD, GSH and MDA in serum and gastric tissues of mice.

**Serum Levels**	**SOD (U/mL)**	**GSH (mg/L)**	**MDA (nmoL/mL)**
Normal	135.27 ± 6.39 ^a^	15.03 ± 1.12 ^a^	10.08 ± 0.25 ^e^
Control	51.07 ± 3.65 ^e^	3.57 ± 0.32 ^e^	69.71 ± 4.48 ^a^
Ranitidine	118.75 ± 3.56 ^b^	13.05 ± 0.44 ^b^	17.66 ± 1.82 ^d^
PRLT-L	79.82 ± 3.41 ^d^	7.71 ± 0.47 ^d^	47.52 ± 3.28 ^b^
PRLT-H	107.51 ± 4.46 ^c^	11.20 ± 0.39 ^c^	25.17 ± 3.59 ^c^
**Gastric Tissue Levels**	**SOD (U/mgprot)**	**GSH (mg/gprot)**	**MDA (nmol/mgprot)**
Normal	218.65 ± 21.08 ^a^	7.08 ± 0.26 ^a^	2.63 ± 0.31 ^e^
Control	89.37 ± 8.35 ^e^	0.97 ± 0.12 ^e^	10.11 ± 0.42 ^a^
Ranitidine	199.59 ± 9.36 ^b^	5.89 ± 0.14 ^b^	3.42 ± 0.28 ^d^
PRLT-L	132.10 ± 10.22 ^d^	2.39 ± 0.19 ^d^	7.32 ± 0.26 ^b^
PRLT-H	171.26 ± 8.97 ^c^	5.12 ± 0.12 ^c^	4.31 ± 0.33 ^c^

Values presented are the mean ± standard deviation (N = 10/group). ^a–^^e^ Mean values with different letters in the same row are significantly different (*p* < 0.05) according to Duncan’s multiple-range test. PRLT-L: 100 mg/kg polyphenols of raw Liubao tea treatment; PRLT-H: 200 mg/kg polyphenols of raw Liubao tea treatment; Ranitidine: 50 mg/kg ranitidine treatment. SOD: superoxide dismutase; GSH: glutathione; and MDA: malondialdehyde.

**Table 7:** Gradient elution conditions for mobile phase.

No.	Time (min)	Flow Rate (mL/min)	%A	%B
1	0.0	0.6	90.0	10.0
2	6.5	0.6	81.5	18.5
3	27.5	0.6	64.5	35.5
4	32.5	0.6	0.0	100.0
5	42.5	0.6	0.0	100.0

**Table 8:** Sequences of reverse transcription-polymerase chain reaction primers were used in this study.

Gene Name	Sequence
Cu/Zn-SOD	Forward: 5′-GAA GAG AGG CAT GTT GGA GA-3′
	Reverse: 5′-CCA ATT ACA CCA CGA GCC AA-3′
Mn-SOD	Forward: 5′-TTC AAT AAG GAG CAG GGA C-3′
	Reverse: 5′-CAG TGT AAG GCT GAC GGT TT-3′
CAT	Forward: 5′-GGA GGC GGG AAC CCA ATA G-3′
	Reverse: 5′-GTG TGC CAT CTC GTC AGT GAA-3′
nNOS	Forward: 5′-ATG TCC TCA AAG CCA TCC AG-3′
	Reverse: 5′-ACT CAG ATC TAA GGC GGT TG-3′
eNOS	Forward: 5′-TGT CTG CGG CGA TGT CAC T-3′
	Reverse: 5′-CAT GCC GCC CTC TGT TG-3′
iNOS	Forward: 5′-CAG CTG GGC TGT ACA AAC CTT-3′
	Reverse: 5′-CAT TGG AAG TGA AGC GTT TGG-3′
COX-2	Forward: 5′-TTA AAA TGA GAT TGT CCG AA-3′
	Reverse: 5′-AGA TCA CCT CTG CCT GAG TA-3′
GAPDH	Forward: 5′-TGC ACC ACC AAC TGC TTA G-3′
	Reverse: 5′-GAT GCA GGG ATG ATG TTC-3′

Cu/Zn-SOD, copper/zinc superoxide dismutase; Mn-SOD, manganese superoxide dismutase; CAT, catalase; nNOS, neuronal nitric oxide synthase; eNOS, endothelial nitric oxide synthase; iNOS, inducible nitric oxide synthase; COX-2, cyclooxygenase-2; and GAPDH, glyceraldehyde-3-phosphate dehydrogenase.
